# The Spectrum of Psychiatric Comorbidity in Individuals With Inflammatory Bowel Disease

**DOI:** 10.1093/crocol/otaf035

**Published:** 2025-05-14

**Authors:** Kianna Cadogan, Ruth Ann Marrie, Lesley A Graff, Renee El Gabalawy, Murray W Enns, James M Bolton, Jitender Sareen, Charles N Bernstein

**Affiliations:** Department of Internal Medicine, Max Rady College of Medicine, Rady Faculty of Health Sciences, University of Manitoba, Winnipeg, Canada; University of Manitoba IBD Clinical and Research Centre, Winnipeg, Canada; University of Manitoba IBD Clinical and Research Centre, Winnipeg, Canada; Department of Medicine, Faculty of Medicine, Dalhousie University, Halifax, Canada; University of Manitoba IBD Clinical and Research Centre, Winnipeg, Canada; Department of Clinical Health Psychology, Max Rady College of Medicine, Rady Faculty of Health Sciences, University of Manitoba, Winnipeg, Canada; University of Manitoba IBD Clinical and Research Centre, Winnipeg, Canada; Department of Clinical Health Psychology, Max Rady College of Medicine, Rady Faculty of Health Sciences, University of Manitoba, Winnipeg, Canada; University of Manitoba IBD Clinical and Research Centre, Winnipeg, Canada; Department of Psychiatry, Max Rady College of Medicine, Rady Faculty of Health Sciences, University of Manitoba, Winnipeg, Canada; University of Manitoba IBD Clinical and Research Centre, Winnipeg, Canada; Department of Psychiatry, Max Rady College of Medicine, Rady Faculty of Health Sciences, University of Manitoba, Winnipeg, Canada; University of Manitoba IBD Clinical and Research Centre, Winnipeg, Canada; Department of Psychiatry, Max Rady College of Medicine, Rady Faculty of Health Sciences, University of Manitoba, Winnipeg, Canada; Department of Internal Medicine, Max Rady College of Medicine, Rady Faculty of Health Sciences, University of Manitoba, Winnipeg, Canada; University of Manitoba IBD Clinical and Research Centre, Winnipeg, Canada

**Keywords:** psychiatric comorbidity, inflammatory bowel disease, SCID-IV, major depressive disorder, anxiety disorder

## Abstract

**Background:**

Research on psychiatric comorbidity in inflammatory bowel disease (IBD) has focused mostly on anxiety and depression. This study aimed to describe the spectrum of psychiatric disorders experienced by individuals with IBD and their overlap.

**Methods:**

Participants were enrolled in a prospective 3-year longitudinal study that assessed psychiatric comorbidity in immune-mediated inflammatory disease. Lifetime prevalence of psychiatric comorbidity was assessed using the Structured Clinical Interview for DSM-IV Disorders (SCID-IV), as the DSM-IV was the prevailing classification at the time of study design. Diagnosis was aligned with DSM-5 categorization where possible with available data. Psychiatric burden was categorized as no psychiatric conditions, 1, 2 or 3 or more psychiatric conditions.

**Results:**

Of 154 IBD participants (62%female, 63% Crohn’s disease) 57% had at least one psychiatric comorbidity with 27% having >1 psychiatric diagnosis. The prevalence was major depressive disorder (MDD, 41.7%), anxiety disorders (39.6%; grouped as per DSM-5), substance use disorder (SUD, 16.2%), posttraumatic stress disorder (5.3%), obsessive-compulsive disorder (4.9%), and bipolar disorder (2.0%). Of participants with MDD and a comorbid psychiatric disorder, nearly half had SUD. Of those with >1 psychiatric disorder >70% had MDD and a comorbid anxiety disorder. Persons with ≥1 psychiatric comorbidity were more likely to be current smokers (*P* < .001) and to have higher IBD disease activity scores (*P* = .005) than those without a psychiatric comorbidity.

**Conclusions:**

Over half of adults with IBD had >1 diagnosed psychiatric comorbidity from a range of 10 different psychiatric disorders identified. Further research should assess the temporal relationship of IBD and the various psychiatric presentations to better understand the trajectory of co-occurrence, and therapy which may concurrently address the psychiatric disorder and the IBD.

## Introduction

Individuals with inflammatory bowel disease (IBD) have an elevated prevalence of psychiatric disorders as compared to those without IBD.^1–3^ In IBD, psychiatric comorbidity is associated with higher health care utilization^4^ and lower quality of life.^5^ Furthermore, it can adversely affect disease progression, may be associated with more complications and may reduce the effectiveness of IBD treatment.^6^ In a multiinstitution cohort study, comorbid depression and anxiety were associated with more colonoscopies, increased use of immunomodulators, and increased risk of requiring surgery in patients with Crohn’s disease.^7^ Moreover, patients with IBD and comorbid anxiety and depression have lower rates of treatment adherence which can also lead to more difficult disease control.^5^ Hence, treating anxiety and depression in patients with IBD aids in the management of IBD.^8^

Most studies on psychiatric comorbidity in IBD have focused on anxiety and depression,^3,5^ variably examining symptoms or diagnostic categories. However, the literature is limited regarding the full scope of psychiatric comorbidities in IBD, with much less known about the prevalence of other psychiatric disorders in IBD, including substance use disorder (SUD),^9^ or their co-occurrence patterns in IBD, despite relevance for psychiatric comorbidity and IBD management. It is poorly understood whether individuals with IBD who have multiple comorbid psychiatric disorders have different characteristics than those with only one psychiatric disorder. Awareness of such differences could inform clinicians’ approaches to screening for psychiatric disorders. This study aimed to describe the spectrum of psychiatric disorders experienced by individuals with IBD and their overlap. The study also aimed to compare characteristics of individuals with one psychiatric disorder to those who have multiple psychiatric disorders, to better inform care.

## Methods

### Data Source and Study Population

The data for this study were drawn from a prospective 3-year longitudinal study in Manitoba, Canada that assessed current and lifetime psychiatric comorbidity in immune-mediated inflammatory disease, inclusive of adults with multiple sclerosis, IBD, and rheumatoid arthritis.^10^

The present cross-sectional analysis focused on the participants with IBD and used data from the psychiatric assessment interview, which was completed only at the baseline visit. Further details are provided in the study protocol.^10^ General strategies used to recruit participants included placing posters in clinics and hospitals, use of social media outlets and self-help groups for mental health concerns. Additionally, to specifically recruit participants with IBD, research assistants approached individuals diagnosed with IBD who attended tertiary outpatient gastroenterology clinics at the province’s largest trauma and tertiary hospital. Research assistants used a standardized script when directly contacting these individuals. Potential participants were also contacted by email through a population-based IBD registry that includes nearly half of Manitoba’s IBD population.^4^ All participants enrolled in the study were required to be over 18 years of age, able to provide informed consent to participate in the study and have basic knowledge of the English language to participate. Inflammatory bowel disease diagnosis was confirmed by chart review and verification with the participant’s gastroenterologist. Ethics approval was obtained from the University of Manitoba Health Research Ethics Board.

### Participant Characteristics

Participants reported their sex, date of birth, education, annual household income, smoking status, and physical comorbidities. Sex was defined as male or female. For this analysis, age was categorized as 18-30, >30-50, >50-60, >60 years. Education was categorized as less than high school, high school diploma, postsecondary education, or advanced degree. Annual household income was captured as <$15 000, $15-49 000, $50-100 000, >$100 000 or do not wish to answer. For this analysis, smoking status was defined as active smoker or ex-smoker/nonsmoker. Data were collected regarding whether participants were ever smokers (defined as ≥100 lifetime cigarettes) and when ex-smokers quit smoking.^10^ Physical comorbidity was assessed by using a self-reported medical history, subsequently verified by medical records review, and categorized as 0, 1, 2, ≥3 physical comorbidities, with common examples including hypertension, dyslipidemia, and osteoarthritis.

### Psychiatric Disorders

Lifetime psychiatric comorbidity was assessed using the Structured Clinical Interview for Diagnostic Statistical Manual of Mental Disorders (DSM)-IV Disorders (SCID-IV).^11^ The SCID-IV was used for interviews because DSM-IV was the prevailing classification at the time the multiyear study was designed. However, questions were added to assess the posttraumatic stress disorder (PTSD) using DSM-5 criteria. The interviews were conducted by individuals trained by a registered clinical health psychologist with experience with the SCID. The disorders assessed in the interview were major depressive disorder (MDD), agoraphobia, generalized anxiety disorder, social phobia, specific phobia, panic disorder, obsessive-compulsive disorder, bipolar disorder, and SUD (including drug and alcohol use disorder), and in addition PTSD. In keeping with the DSM-5 classification, obsessive-compulsive disorder and PTSD were not grouped as anxiety disorders and were considered separately. Substance use disorders were only assessed as lifetime, not current.

### IBD Characteristics

Symptomatic disease activity was measured using the Powell Tuck Index for Ulcerative Colitis^12^ and the Harvey Bradshaw Disease Activity Index for Crohn’s Disease (HBI).^13^ For each of these validated clinical indices, active symptomatic disease activity was defined as a score ≥5. Data were also gathered regarding the age of onset and duration of disease.

### Analysis

The characteristics of the study population were summarized using descriptive statistics. For completeness we report current prevalence of each psychiatric disorder, but we focused the analysis on lifetime prevalence. The lifetime prevalence of each psychiatric disorder was described, as well as the overlap between multiple conditions. Psychiatric burden was categorized as no psychiatric conditions, one psychiatric condition, 2 psychiatric conditions, or 3 or more psychiatric conditions. Different anxiety disorders were not grouped for this categorization. The characteristics of the groups, according to number of psychiatric conditions, were analyzed. Comparisons were made using a chi-square test as well as a test for linear trend.

The IBM SPSS Statistics software Version 29.0.1.0 (IBD Corp.) was used for data analysis.

## Results

### Participant Characteristics

Two-thirds of participants were female (Table 1). Over half of participants had achieved some level of postsecondary education. Nearly 70% had an annual household income over fifty thousand dollars. Overall, two-thirds of participants had Crohn’s disease. Over half of participants had a disease activity score <5 (57%). Most participants had IBD onset in adulthood.

**Table 1.** Comparison based on number of psychiatric disorders.

	No disorders	One disorder	Two disorders	Three or more disorders	Total	*P*-value
**Type of IBD, *n* (%)**						.46
Crohn’s disease	66 (61.1)	46 (63.0)	16 (53.3)	26 (72.2)	154 (62.3)	
Ulcerative colitis	42 (38.9)	27 (37.0)	14 (46.7)	10 (27.8)	93 (37.7)	
**Sex, *n* (%)**						.30
Male	38 (35.2)	29 (39.7)	14 (46.7)	9 (25.0)	90 (36.4)	
Female	70 (64.8)	44 (60.3)	16 (53.3)	27 (75.0)	157 (63.6)	
**Age, *n* (%)**						.62
18-30	19 (17.6)	13 (17.8)	6 (20.0)	6 (16.7)	44 (17.8)	
>30-50	33 (30.6)	25 (34.2)	14 (46.7)	17 (47.2)	89 (36.0)	
>50-60	27 (25.0)	17 (23.3)	7 (23.3)	6 (16.7)	57 (23.1)	
>60	29 (26.9)	18 (24.7)	3 (10.0)	7 (19.4)	57 (23.1)	
**Education, *n (*%)**						.72
Less than High School Diploma	4 (3.7)	2 (2.7)	0 (0)	2 (5.6)	8 (3.2)	
High School Diploma	27 (25.0)	19 (26.0)	9 (30.0)	13 (36.1)	68 (27.5)	
Postsecondary Education	66 (61.1)	42 (57.5)	15 (50.0)	16 (44.4)	139 (56.3)	
Other	11 (10.2)	10 (13.7)	6 (20.0)	5 (13.9)	32 (13.0)	
**Annual household income, *n* (%)**						.07
<$15 000	3 (2.8)	5 (6.8)	3 (10.0)	4 (11.1)	15 (6.1)	
$15 000-49 999	12 (11.1)	20 (27.4)	4 (13.3)	8 (22.2)	44 (17.8)	
$50 000-100 000	41 (38.0)	31 (42.5)	15 (50.0)	16 (44.4)	103 (41.7)	
>$100 000	39 (36.1)	14 (19.2)	7 (23.3)	7 (19.4)	67 (27.1)	
Prefer not to answer	13 (12.0)	3 (4.1)	1 (3.3)	1 (2.8)	18 (7.3)	
**Smoking status, *n* (%)** ^a^						<.001
Smoker	6 (5.6)	18 (24.7)	8 (26.7)	10 (27.8)	42 (17.0)	
nonsmoker/Ex-smoker	102 (94.4)	55 (75.3)	22 (73.3)	26 (72.2)	205 (83.0)	
**Physical comorbidity, *n* (%)**						.12
0	43 (39.8)	19 (26.0)	10 (33.3)	11 (30.6)	83 (33.6)	
1	27 (25.0)	19 (26.0)	2 (6.7)	10 (27.8)	58 (23.5)	
2	15 (13.9)	10 (13.7)	9 (30.0)	7 (19.4)	41 (16.6)	
3 or more	23 (21.3)	25 (34.2)	9 (30.0)	8 (22.2)	65 (26.3)	
**Powell Tuck Score/Harvey Bradshaw Score, *n* (%)** ^a^						.006
<5	72 (66.7)	41 (56.2)	15 (50.0)	13 (36.1)	141 (57.1)	
≥5	31 (28.7)	31 (42.5)	15 (50.0)	19 (52.8)	96 (38.9)	
Other/Not available	5 (4.6)	1 (1.4)	0 (0)	4 (11.1)	10 (4.0)	
**Age at onset (years), *n* (%)**						.99
<17	15 (13.9)	9 (12.3)	3 (10.0)	5 (13.9)	32 (13.0)	
17-40	73 (67.6)	48 (65.8)	20 (66.7)	23 (63.9)	164 (66.4)	
>40	20 (18.5)	16 (21.9)	7 (23.3)	8 (22.2)	51 (20.6)	
**Total**	108 (43.7)	73 (29.6)	30 (12.1)	36 (14.6)	247	

^a^Signifies a statistically significant result.

Most participants did not currently smoke (83%); however, close to two-thirds had at least one physical comorbidity, with the most common presentations being hypertension, dyslipidemia, and osteoarthritis.

### Psychiatric Comorbidity

**Figure 1.**
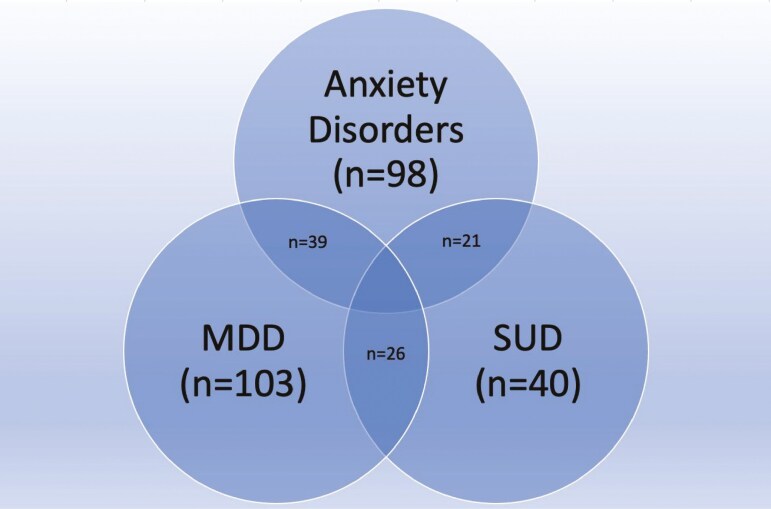
Spectrum of psychiatric disorders. Abbreviations: Anxiety disorders = agoraphobia + generalized anxiety disorder + social phobia + specific phobia + panic disorder; MDD, major depressive disorder; SUD, substance use disorder.

Over half of participants (57%) had at least one diagnosed psychiatric condition in their lifetime with 27% having more than one. Major depressive disorder was the most prevalent psychiatric disorder (41.7%, *n* = 103). When the 5 anxiety disorders were grouped together (ie, agoraphobia, generalized anxiety disorder, social phobia, simple phobia, and panic disorder), anxiety disorders were the second most prevalent psychiatric presentation (39.6%, *n* = 98), followed by SUD (16.2%, *n* = 40) (Figure 1). The least prevalent psychiatric disorder was bipolar disorder (2.0%, *n* = 5) (Table 2).

**Table 2.** Frequency of lifetime psychiatric disorders.

Psychiatric disorder	Lifetime, *n* (%)	Current, *n* (%)
Any psychiatric disorder	139 (56.3)	58 (23.5)^c^
Major depressive disorder	103 (41.7)	21 (8.5)
Anxiety disorders	98 (39.8)	67 (27.1)
Generalized anxiety disorder	24 (9.7)	14 (5.7)
Social phobia	32 (13.0)	27 (10.9)
Specific phobia	19 (7.7)	17 (6.9)
Panic disorder	12 (4.9)	3 (1.2)
Agoraphobia	11 (4.5)	6 (2.4)
Obsessive-compulsive disorder	12 (4.9)	5 (2.0)
Posttraumatic stress disorder	13 (5.3)	8 (3.2)
Bipolar disorder^a^	5 (2.0)	1 (0.4)
Substance use disorder^b^	40 (16.2)	–

^a^For bipolar disorder, current indicates met symptomatic criteria for manic, hypomanic, mixed, or major depressive episode in past month.

^b^Substance use disorders only assessed as lifetime, not current. **.

^c^Excludes substance use disorders.

For the participants with more than one psychiatric disorder **ever** some combinations of psychiatric disorders occurred more often together. Of our participants with MDD, 53 (51.5%) had a comorbid psychiatric disorder, of which nearly half (49.1%) of these being a SUD. Among people with IBD and a comorbid SUD, 81.3% of those had comorbid depression. Of all persons with MDD and at least one other psychiatric disorder, in over 70% the other disorder was an anxiety disorder. Over 60% of those with SUD with a comorbid psychiatric disorder had an anxiety disorder (Table 3).

**Table 3.** Co-occurring psychiatric disorders in participants with multiple psychiatric disorders.

Psychiatric disorder *n*, (%)	MDD (*n* = 103)	DSM-V anxiety disorders (*n* = 98)	OCD (*n* = 12)	PTSD (*n* = 13)	Bipolar disorder (*n* = 5)	SUD (*n* = 40)	Total
MDD	X	39 (73.6)	10 (18.9)	13 (24.5)	4 (7.5)	26 (49.1)	53
DSM-V Anxiety Disorders	39 (79.6)	X	7 (14.3)	12 (24.5)	2 (4.1)	21 (42.9)	49
OCD	10 (83.3)	7 (58.3)	X	4 (33.3)	1 (8.3)	5 (41.7)	12
PTSD	13 (92.9)	12 (85.7)	4 (28.6)	X	1 (7.1)	5 (35.7)	14
BPD	4 (100.0)	2 (50.0)	1 (25.0)	1 (25.0)	X	2 (50.0)	4
SUD	26 (81.3)	21 (65.6)	5 (15.6)	5 (15.6)	2 (6.3)	X	32

Abbreviations: BPD, bipolar disorder; DSM-V anxiety disorders, agoraphobia + generalized anxiety disorder + social phobia + specific phobia + panic disorder; MDD, major depressive disorder; OCD, obsessive-compulsive disorder; PTSD, posttraumatic stress disorder; SUD, substance use disorder.

### Factors Associated with Psychiatric Comorbidity

Participants with one or more lifetime psychiatric comorbidities were more likely to be current smokers than those without a psychiatric comorbidity [*X*^2^ (3, *N* = 247) = 18.006, *P* < .001]. Participants with a psychiatric comorbidity were more likely to have higher IBD disease activity scores than participants without a psychiatric disorder. Participants with multiple psychiatric disorders were more likely to report active IBD than participants with only one psychiatric disorder and those with no psychiatric disorders [*X*^2^ (6, *N* = 247) = 17.913, *P* = .005]. Other characteristics did not differ between groups (Table 1).

There was a linear association with increased number of psychiatric comorbidities and being an active smoker [linear by linear association 13.269 (*P* < .001)], and higher IBD activity [linear by linear association 5.095 (*P* = .024)]. Other characteristics that were analyzed showed no significant linear association (Table 1).

## Discussion

In this cross-sectional analysis of psychiatric comorbidity patterns in individuals with IBD, we found that participants with IBD in this study experienced a relatively broad range of psychiatric comorbidities over their lifetime, most often including MDD, anxiety disorders, and SUD. Previously we reported that the prevalence of current suicidality based on the SCID was 2%, and based on the Patient Health Questionnaire-9 was 9.7% in this IBD population.^14^ Over 1 in 2 participants had at least one psychiatric disorder and more than 1 in 4 had more than one psychiatric disorder. Major depressive disorder and anxiety disorders very commonly occurred together. Participants with SUD also frequently had comorbid depression or anxiety disorders. Participants with one or more psychiatric comorbidities were more likely to be active smokers compared to participants without a psychiatric disorder, and those with a lifetime prevalence of multiple psychiatric disorders were more likely to have active symptomatic IBD.

While most literature regarding psychiatric comorbidity in IBD has focused on anxiety and depression and its implications for management in IBD,^1,3,5,15^ this study highlights the frequency with which multiple psychiatric disorders co-exist in IBD. Historically, psychiatric disorders have been diagnosed categorically based on a constellation of type of symptoms, number of symptoms, and associated distress. Further, symptoms can overlap between disorder categories. Consequently, a person who meets criteria for one disorder often meets criteria for other psychiatric disorders as we observed, and studies that focus on a single disorder may not be representative of the whole population. These observations have highlighted the importance of developing novel therapeutic approaches based on underlying neurobiology.^16^

The literature supports treating both psychiatric disorders and inflammatory disease together given their bi-directional relationship, although there have been limited studies assessing effectiveness of specific medication therapies for treating psychiatric disorders in this population.^17^ One retrospective study showed that treatment of comorbid anxiety and depression in patients with IBD with antidepressants reduced the need for corticosteroids, relapses in disease activity, and reduced need for investigations.^18^ A systematic review in 2017 that included 43 studies concluded that psychotherapy as well as antidepressants had benefits regarding IBD activity as well as health-related quality of life, supporting psychiatric treatment in this population.^8^

While the literature supports treating both the inflammatory disorder and the psychiatric disorder, less is known regarding optimal management of multiple co-occurring psychiatric disorders. A systematic review in 2020 reviewed the existing guidelines for management of concurrent psychiatric disorders.^19^ The review emphasized that there was very limited specific evidence for management of concurrent psychiatric disorders, but that current guidelines generally supported combining treatment regimens for each individual psychiatric disorder. However, it noted the shortcoming of this depending on the combination of psychiatric disorder, and thus an individual, tailored approach is necessary related to the psychiatric disorder presentations.^19^ Further, this review was without consideration of other major health concerns such as a chronic inflammatory disease. Thus, recognizing when individuals have multiple psychiatric disorders is important as it may significantly change the management strategy for the psychiatric disorder and ultimately the management of their IBD. For the clinician managing the person with IBD, this means that when concern arises about a possible psychiatric disorder a comprehensive assessment is required to ensure appropriate diagnosis(es) and management. This is likely to require engagement of a mental health specialist. As one example, there has been a movement to treat addictions concurrently with other mental disorders to reduce the silos that have historically separated the clinical approach to managing these conditions.^20^

The rates of the psychiatric conditions in our sample of individuals with IBD are important to examine relative to the general population. Recent Canadian data indicate prevalence estimates of generalized anxiety disorder were 5.2%, MDD was 7.6% and bipolar disorder was 2.1% in our Canadian population aged 15 and older.^21^ A cross-sectional population-based study in Australia studying the prevalence of depression and anxiety in individuals with chronic diseases found that the highest prevalence of depression and/or anxiety was in persons with hypertension (34.7%), followed by persons with arthritis (33.3%), then chronic back/neck pain (31.9%).^22^ This compares to the prevalence of anxiety disorders (49.8%) and MDD (41.7%) among persons with IBD in our study. This further indicates that the prevalence of psychiatric disorders is higher in many chronic diseases compared to the general population.^22^ Our group has previously reported that social phobia is comparably present in multiple sclerosis (10.2%) and rheumatoid arthritis (17%), as in IBD (13%).^23^ We also reported that SUD had a prevalence of 14.9% in rheumatoid arthritis, compared to 16.2% in the IBD population.^24^

Although the literature regarding multiple psychiatric comorbidities in relation to chronic diseases is sparse, there was a cross-sectional observational study in 2023 that studied the prevalence of multiple psychiatric comorbidities in hospitalized patients with Parkinson’s disease and atypical Parkinsonian syndromes.^25^ The study found that of their 110 study participants 38.0% of those with Parkinson’s disease and 28.2% of those with atypical Parkinsonian syndrome had multiple psychiatric comorbidities. Additionally, they found that reported caregiver stress was higher in those with multiple psychiatric comorbidities.^25^ Elsewhere, a study assessing comorbid MDD and panic disorder found that those with both psychiatric comorbidities had a higher risk of suicide, more severe psychiatric symptoms, and harder to treat psychiatric disorders compared to having just one psychiatric disorder.^26^ These findings suggest that the presence of multiple psychiatric comorbidities may be associated with disease complications both with the chronic disease as well as the psychiatric disorder. This could relate to our finding that individuals with multiple psychiatric disorders were more likely to report active disease than individuals with only one psychiatric disorder.

We found that participants with psychiatric disorders were more likely to have active symptomatic inflammatory disease. Previous studies have observed increased disease activity in patients with IBD and comorbid depression and anxiety. Most of these studies also used subjective measures of disease activity,^27^ however, others have used objective clinical measures. Additionally, some studies have identified that this population may have irritable bowel symptoms that may be mistaken as IBD symptoms.^16,28^ In a longitudinal study examining IBD disease activity as well as psychiatric symptoms in individuals with IBD and comorbid psychiatric disorders, fecal calprotectin was used to identify inflammatory disease activity. That study found that the need for steroids in disease activity, marked by elevated fecal calprotectin, was higher in those who also had active mental health symptoms, and concluded that managing symptoms of mental health disorders should be considered a treatment target in individuals with IBD.^29^ While not assessed in our study, insomnia is also common in patients with IBD and has been associated with clinically active IBD, depression, and anxiety.^30^ The temporal link between IBD and psychiatric disorders and disease activity is not well understood, and they likely interact in multiple ways.^16^

This study has many strengths, most notably the careful evaluation of a range of mental disorders through semistructured diagnostic interview by trained reviewers, confirmed medical diagnosis of IBD, and validated clinical indices for disease activity. Limitations include that we were not able to define the temporal association between the psychiatric disorders and disease activity, we did not assess psychotic disorders, did not have inflammatory markers for disease activity such as fecal calprotectin to determine IBD severity, or psychiatric severity from the study database.

We used the SCID-IV, which is based on the DSM-IV classifications, due to the timing of the study design in relation to the DSM-IV as the prevailing system, noting the study was a multiyear, longitudinal design. While we were able to add questions to assess the DSM-5 criteria for PTSD, and could consider PTSD and obsessive-compulsive disorder separately (ie, not as anxiety disorders), to align with the DSM-5 framework, the use of the SCID-IV is acknowledged as a limitation for comparison with current diagnostic categories.^11^ Eating disorders were not evaluated in our study, and can be difficult to assess in the context of gastrointestinal disease because irregularities in eating can cause gastrointestinal symptoms. Eating disorders have commonly been found to precede gastrointestinal disorders, and to be most highly associated with disorders of the brain–gut axis including functional and motility presentations.^31,32^ Future studies, including data on the co-existence of eating disorders with other psychiatric disorders in IBD would provide more insight into this.

## Conclusions

Over half of participants with IBD had at least one psychiatric comorbidity and the spectrum of psychiatric disorders included 10 different diagnoses. Having more than one psychiatric disorder was associated with higher disease activity. This study should alert practitioners to the spectrum of psychiatric diagnoses in persons with IBD which may affect management. Further research should assess the temporal relationship of IBD and the various psychiatric comorbidities to better understand what preventative and therapeutic measures may be helpful for both the psychiatric disorder and the IBD.

## Data Availability

The datasets presented in this article are not readily available because some participants did not agree to data sharing. Components of the datasets may be made accessible to qualified investigators with the appropriate ethical approvals and data use agreements upon request. Requests to access the datasets should be directed to rmarrie@dal.ca.
